# The impact of psychological abuse by an intimate partner on the mental health of pregnant women

**DOI:** 10.1111/j.1471-0528.2007.01593.x

**Published:** 2008-02

**Authors:** A Tiwari, KL Chan, D Fong, WC Leung, DA Brownridge, H Lam, B Wong, CM Lam, F Chau, A Chan, KB Cheung, PC Ho

**Affiliations:** a Department of Nursing Studies, LKS Faculty of Medicine The University of Hong KongHong Kong, China; b Department of Social Work and Social Administration, The University of Hong KongHong Kong, China; c Department of Obstetrics and Gynaecology, Kwong Wah HospitalHong Kong, China; d Department of Family Social Sciences, Arthur V. Mauro Centre for Peace and Justice, University of ManitobaWinnipeg, Canada; e Department of Obstetrics and Gynaecology, Pamela Youde Nethersole Eastern HospitalHong Kong, China; f Department of Obstetrics and Gynaecology, Princess Margaret HospitalHong Kong, China; g Department of Obstetrics and Gynaecology, Queen Elizabeth HospitalHong Kong, China; h Department of Obstetrics and Gynaecology, United Christian HospitalHong Kong, China; i Department of Obstetrics and Gynaecology, Tuen Mun HospitalHong Kong, China; j Department of Obstetrics and Gynaecology, LKS Faculty of Medicine, The University of Hong KongHong Kong, China

**Keywords:** Intimate partner violence, pregnancy, psychological abuse

## Abstract

**Objective:**

The objective of this first population-based study in Hong Kong was to assess the impact of psychological abuse by an intimate partner on the mental health of pregnant women.

**Design:**

Survey.

**Setting:**

Antenatal clinics in seven public hospitals in Hong Kong.

**Population:**

Three thousand two hundred and forty-five pregnant women.

**Methods:**

The Abuse Assessment Screen (AAS) and demographic questionnaires were administered face-to-face at 32–36 weeks of gestation. At 1 week postpartum, the AAS, Edinburgh Postnatal Depression Scale and SF-12 Health Survey were administered by telephone.

**Main outcome measures:**

Intimate partner violence, postnatal depression and health-related quality of life.

**Results:**

Two hundred and ninety six (9.1%) of the participants reported abuse by an intimate partner in the past year. Of those abused, 216 (73%) reported psychological abuse only and 80 (27%) reported physical and/or sexual abuse. Forty six (57.5%) in the physical and/or sexual abuse group also reported psychological abuse. Women in the psychological abuse only group had a higher risk of postnatal depression compared with nonabused women (adjusted OR: 1.84, 95% CI: 1.12–3.02). They were also at a higher risk of thinking about harming themselves (adjusted OR: 3.50, 95% CI: 1.49–8.20) and had significantly poorer mental health-related quality of life (*P* < 0.001). The higher risks of postnatal depression and thinking of harming themselves were not observed in the physical and/or sexual abuse group although significantly poorer mental health-related quality of life (*P* < 0.001) was observed.

**Conclusions:**

Psychological abuse by an intimate partner against pregnant women has a negative impact on their mental health postdelivery. Furthermore, psychological abuse in the absence of physical and/or sexual abuse can have a detrimental effect on the mental health of abused women. The findings underscore the importance of screening pregnant women for abuse by an intimate partner and the need for developing, implementing and evaluating interventions to address psychological abuse.

*Please cite this paper as*: Tiwari A, Chan K, Fong D, Leung W, Brownridge D, Lam H, Wong B, Lam C, Chau F, Chan A, Cheung K, Ho P. The impact of psychological abuse by an intimate partner on the mental health of pregnant women. BJOG 2008;115:377–384.

## Introduction

Intimate partner violence during pregnancy has been well researched with numerous studies reporting on the prevalence of violence against pregnant women and the association between violence and pregnancy outcomes.^1^,^2^ Much of the research, however, has been focused on physical abuse.

Relatively little attention has been given to psychological abuse of women, despite the findings that after statistically controlling for the effects of physical abuse, psychological abuse has been associated with adverse mental health outcomes including depressive symptomatology.^3^ Research on psychological abuse and its effect on the health and wellbeing of pregnant women has similarly been limited despite concerns expressed about the association between verbal abuse and adverse infant outcome.^4^ A recent study found that women who experienced psychological abuse by their intimate partners during pregnancy had higher levels of depressive symptoms compared with nonabused women and further recommended an examination of its effect on abused women's mental health after delivery.^5^

In a prospective study involving a group of pregnant Chinese women in a teaching hospital in Hong Kong, psychological abuse was significantly related to postnatal depression.^6^ The findings affirmed the need to conduct further research to examine the effects of psychological abuse on the mental health of pregnant women using multiple sites,^7^ well-trained and skilled clinicians,^1^ and measures of psychological abuse.^8^ The purpose of this study was to extend previous research on the effect of psychological abuse on the mental health of pregnant women. We hypothesised that, similar to the study by Leung *et al*.,^6^ psychological abuse against pregnant women would adversely impact on their mental health postdelivery. In our test of the hypothesis, we went beyond measurement of postnatal depression to assess the impact of psychological abuse on the mental health component of the health-related quality of life.

## Methods

We conducted the first population-based survey in Hong Kong involving obstetrics and gynaecology departments in seven hospitals, which collectively have about 34 000 deliveries per year. The study was conducted between 1 July 2005 and 30 April 2006. During this period, all women (≥18 years old) between 32 and 36 weeks of pregnancy were eligible for the study. The exclusion criteria were: women who were unable to be questioned without the presence of a family member or who were not competent to give informed consent.

We defined intimate partner violence as a pattern of coercive control. This included the use of physical violence, sexual assault and/or emotional abuse or the use of threats or coercive tactics against a woman by her current or former intimate partner who may be a current or former spouse, cohabiting partner, boyfriend, or dating partner.^9^ As previous local studies^6^,^10^ conducted in similar settings have revealed that intimate partner violence in Chinese couples is predominantly psychological in nature, ‘violence’ is therefore broadly defined in this study: it is not confined to violence in the sense of hitting, kicking or similar but also includes the more subtle form of abuse such as psychological abuse.

The Abuse Assessment Screen (AAS) was used to detect the prevalence of intimate partner violence. The AAS was developed by the Nursing Research Consortium on Violence and Abuse to determine abuse status and perpetrator within a defined period.^11^ In the present study, for the Chinese participants, we used a Chinese version of the AAS. The Chinese AAS addresses both emotional and physical violence separately for all time periods (lifetime, the preceding 12 months and during pregnancy). It differs from the original English AAS that treats emotional and physical violence simultaneously for the lifetime period while focusing on physical violence for the other time periods. Examples of emotional/psychological abuse commonly cited by Chinese women in previous studies^6^,^10^ were also provided to ensure that participants understood what was meant by ‘emotionally hurt’. The Chinese AAS has been validated and has demonstrated satisfactory measurement accuracy and utility for identifying intimate partner violence.^8^ Women who answered ‘yes’ to any of the questions on physical, emotional/psychological or sexual abuse by an intimate partner in the past year, or since becoming pregnant, were considered abused.

Mental health in this study is defined in accordance with the conceptualisation described in the World Health Report 2001.^12^ Mental health in this sense includes subjective wellbeing as well as perceived self-efficacy and is broader than a lack of mental disorders. Mental health and mental functioning are fundamentally interconnected with physical and social functioning. In this study, we measured mental health using the Edinburgh Postnatal Depression Scale (EPDS)^13^ to elicit subjective depressive symptoms and the SF-12 Health Survey (SF-12)^14^ to assess mental, physical and social functioning.

The Chinese version of the EPDS^13^ was used to detect postnatal depression among the Chinese participants. As recommended, the cutoff score of ≥10 was used for screening depressive illness in the Chinese postnatal population.^13^ In addition, the participants’ responses to question 10 of the EPDS were also examined (‘The thought of harming myself has occurred to me: (1) yes, quite often, (2) sometimes, (3) hardly ever, and (4) never.’)

The standard SF-12,^15^ an abbreviated form of the medical outcomes study (MOS) 36-item Short Form Health Survey (SF 36) was used to assess health-related quality of life. The standard SF-12 consists of 12 items grouped under the physical health summary and mental health summary (MCS) scales. The higher the scale score the better the corresponding quality of life. In this study, for the Chinese participants, we used the Chinese (HK)-specific SF-12, which has been validated and found to have satisfactory psychometric properties compared with the standard SF-12.^14^

We used a demographic questionnaire to collect socio-demographic information from the participants based on the known risk factors documented in the literature. The information included nationality, age, education, marital status, number of children, planned pregnancy, employment, family income, indebtness, financial assistance, social support, consumption of alcohol, chronic illness in the family and in-law conflict.

Pregnancy outcome was assessed by chart review and included the information about preterm delivery, mode of delivery, birthweight, Apgar scores and admission to neonatal intensive care unit (ICU).

Each of the seven experienced research nurses received a 2-day training workshop (conducted by the first author). The workshop dealt with ensuring privacy and safety of the participant during data collection, enhancing consistency when using the measuring instruments in face-to-face and telephone interviews, and promoting maternal and infant health by educating abused women about referrals and community resources.

The study was approved by the institutional review board of each of the participating hospitals. During the study period, women attending antenatal clinics in the seven participating hospitals and who met the inclusion criteria were invited to participate in a private area without the presence of their partners. Sampling was consecutive. It was made clear to the women that participation was entirely voluntary and informed consent was ensured. Those who agreed to participate signed a consent form. At their entry to the study, the AAS and the demographic questionnaire were administered face-to-face by one of the research nurses. Women assessed to be ‘abused’ by the AAS were counselled by the research nurse. Each was provided with a wallet-sized card detailing hotlines and community support for abused women if it was safe for her to take home such information. If not, she was provided with the telephone number of the research nurse, disguised as a nurse from the antenatal clinic, which she could call at any time. The research nurse also asked each of the abused women if she wished to call the police and/or to be referred to a medical social worker as per hospital standard policy.

Before the end of the initial interview, the research nurse obtained from the woman some means of contacting her for the postdelivery interview (home and/or mobile telephone number) and established a code for ensuring her safety when calling her in case her partner was nearby.

At 1 week postdelivery, a research nurse contacted the woman by phone. If it was safe for the woman (e.g. her partner was not nearby), the AAS would be administered again. The repeat administration was conducted because some women may not have disclosed their abusive experience when asked the first time but may do so subsequently. The SF-12 and EPDS were administered over the telephone. Before leaving the woman, our research nurse would check the safety of the woman and her baby, and whether she wished to be referred to any resources designed for abused women in the community.

Sample size calculation was based on the estimation of the prevalence of intimate partner violence. For a maximum tolerable error of 1% and an anticipated prevalence of 10%, a total of 3548 subjects were required using a 95% confidence interval.

Prevalence of abuse by an intimate partner in the past year was reported together with its exact 95% confidence interval obtained from a binomial distribution. To examine the effects of demographics, socio-economic status, history of chronic diseases and in-law conflicts on abuse in the past year, the potential factors were first examined individually by conditional logistic regression to account for the extra covariance among subjects within a hospital. Then, stepwise conditional logistic regression analysis was performed to determine independent effects of the factors. Pseudo *R*^2^ of the final model was calculated as 1 − log *L*(*f*)/log *L*(*i*), where log *L*(*f*) is the log-likelihood value of the final model and log *L*(*i*) is the log-likelihood value of the initial model with the constant only.^16^

The impact of abuse by an intimate partner on postnatal depression (measured by EPDS) was examined using conditional logistic regression with adjustment for demographics, socio-economic status, history of chronic diseases and in-law conflict. Additionally, the impact on quality of life (measured by SF-12) was examined using a mixed effects model with a random effect for each hospital and the same adjustment for potential confounding effects.

Additional sensitivity analyses were performed to examine the effect of missing values. First, missing values of a factor were replaced by the predicted values from a regression on other observed factors. Second, missing values of a factor were simply replaced by the mean of the observed values of the factor.

The level of significance was taken as 5% and the Statistical Analysis System, version 9 was used for the statistical analysis.

## Results

A total of 3570 women qualified for the study and were invited to participate, and of these 223 refused to participate and 102 were excluded from the study as they spoke a dialect not understood by the investigators. A total of 3245 women were recruited and completed the investigator-administered, face-to-face interview at the time of recruitment. A telephone interview was conducted at 1 week postdelivery, and 3036 completed the AAS, EPDS and SF-12. Loss of contact and refusal accounted for the 209 women who did not complete the postdelivery telephone interview.

Of the 3245 participants, 296 (9.1%; 95% CI: 8.2%–10.2%) reported that they were abused by their intimate partners in the past year. Of those abused, 216 (73%) reported psychological abuse only (hereafter known as the psychological abuse only group), while 80 (27%) reported physical and/or sexual abuse (hereafter known as the physical and/or sexual abuse group). Among those in the physical and/or sexual abuse group, 46 (57.5%) also reported psychological abuse.

The most frequently cited examples of psychological abuse were ‘shaming in front of friends/family’, ‘put-downs regarding appearance or behaviour’ and ‘ridiculing’. The perpetrator ‘throwing something at her’, ‘pushing her’ and ‘slapping her’ were the most common examples of physical abuse cited by the women. Being afraid of the perpetrator was expressed by 17.6% of those in the psychological abuse only group and 23.8% in the physical and/or sexual abuse group.

The demographic data on the 3245 women are given in Table 1. The unadjusted effects of factors on intimate partner violence are summarised in Table 2. Table 3 shows the factors associated with intimate partner violence in the past year. There were more factors identified when missing values were imputed. Nevertheless, it is generally consistent that women who were in debt, required financial assistance, whose pregnancy was unplanned and who had in-law conflict were more likely to experience intimate partner violence in the past year.

**Table 1:** Demographic characteristics of participants

	Nonabused (*n*= 2949)	Abused (*n*= 296)	Overall(*n*= 3245)
Age (years), mean (SD)	31 (4.7)	31 (5.6)	31 (4.8)
Married or cohabiting	2881 (98)	275 (93)	3156 (97)
Number of years married, mean (SD)	4.7 (3.7)	5 (4.0)	4.7 (3.7)
Nulliparous	1500 (51)	115 (39)	1615 (50)
Number of children (>2)	1205 (41)	148 (50)	1353 (42)
Unplanned pregnancy	983 (33)	162 (55)	1145 (35)
Alcohol user	158 (5)	28 (10)	186 (6)
Education (≤9 years)	656 (22)	80 (27)	736 (23)
Family monthly income below local average (<HK$10,000)	553 (19)	100 (35)	653 (20)
Financial assistance	1278 (43)	162 (55)	1440 (44)
In debt	148 (5)	44 (15)	192 (6)
Woman in paid job	1654 (56)	123 (42)	1777 (55)
Partner in paid job	2899 (99)	282 (98)	3181 (99)
Chronic illness in family	541 (18)	76 (26)	617 (19)
In-law conflict in the past year	152 (6)	35 (15)	187 (7)

Values are presented as *n* (%) unless otherwise indicated.

**Table 2:** Unadjusted analysis of factors associated with intimate partner violence in the past year

Variable	OR	95%CI
Age (years)	0.99	(0.97–1.01)
Spouse age (years)	1.00	(0.98–1.02)
Age difference (spouse participant)	1.01	(0.98–1.03)
Married or cohabiting	0.27	(0.16–0.46)
Number of years married	1.02	(0.99–1.05)
Number of children	1.36	(1.17–1.57)
First pregnancy	0.64	(0.50–0.81)
Unplanned pregnancy	2.41	(1.89–3.07)
Alcohol user	1.76	(1.16–2.69)
Spouse alcohol user	1.60	(1.22–2.09)
Education	0.88	(0.80–0.98)
Spouse education	0.92	(0.83–1.02)
Family monthly income	0.91	(0.89–0.94)
Financial assistance	3.56	(2.31–5.49)
In debt	3.11	(2.16–4.48)
Unemployed	2.02	(1.40–2.91)
Spouse unemployed	2.72	(1.00–7.34)
Chronic illness in family	2.56	(1.59–4.12)
In-law conflict in the past year	2.58	(1.74–3.83)

**Table 3:** Factors associated with intimate partner violence in the past year

	OR	95%CI
Complete record analysis (*n*= 2177; pseudo *R*^2^= 4.9%)		
In debt	2.25	(1.37–3.69)
Financial assistance	2.54	(1.30–4.97)
Unplanned pregnancy	1.81	(1.32–2.49)
In-law conflict	1.49	(1.26–1.77)
**Imputation by predicted values (*n*= 3245; pseudo *R*^2^= 6.9%)**		
Married or cohabiting	0.44	(0.25–0.78)
Number of children	1.21	(1.04–1.41)
In debt	2.27	(1.55–3.34)
Family monthly income	0.96	(0.93–0.99)
Chronic illness in family	2.17	(1.33–3.55)
Unplanned pregnancy	1.79	(1.38–2.33)
In-law conflict	1.35	(1.20–1.52)
**Imputation by mean values (*n*= 3245; pseudo *R*^2^= 6.8%)**		
Married or cohabiting	0.43	(0.24–0.76)
In debt	2.30	(1.57–3.38)
Income	0.96	(0.93–0.99)
Chronic illness in family	2.15	(1.31–3.52)
First pregnancy	0.75	(0.58–0.97)
Unplanned pregnancy	1.85	(1.43–2.40)
In-law conflict	1.34	(1.19–1.51)

Table 4 shows the impact of intimate partner violence on the EPDS scores. Women in the psychological abuse only group had a higher risk of postnatal depression (adjusted OR: 1.84, 95% CI: 1.12–3.02, *P* = 0.016) compared with nonabused women. With the current sample size, the same impact was not observed in the physical and/or sexual abuse group) (adjusted OR: 1.75, 95% CI: 0.84–3.66, *P* = 0.137). Furthermore, compared with nonabused women, those in the psychological abuse only group were at a higher risk of thinking about harming themselves (adjusted OR: 3.50, 95% CI: 1.49–8.20, *P* = 0.004). However, such an impact was not observed in the physical and/or sexual abuse group (adjusted OR: 1.17, 95% CI: 0.20–6.94, *P* = 0.861).

**Table 4:** Impact of intimate partner violence on EPDS scores

Type of abuse	EPDS scores ≥10 (*n*= 2122)		EPDS (Q. 10 ‘Thinking about harming themselves’) (*n*= 2162)	
		
	OR*	95% CI	OR*	95% CI
Psychological only	1.84	(1.12–3.02)	3.50	(1.49–8.20)
Physical and/or sexual	1.75	(0.84–3.66)	1.17	(0.20–6.94)
No abuse	1	—	1	

* Adjusted by demographics, socio-economic status, chronic illness in family, and in-law conflict.

Table 5 shows the impact of intimate partner violence on the SF-12 scores. In terms of physical health, intimate partner violence had no significant impact (*P* > 0.083). In terms of mental health, both psychological abuse only and physical and/or sexual abuse significantly reduced the MCS scores (*P* < 0.001, respectively). Results were essentially similar to the analysis performed after imputation of missing values.

**Table 5:** Impact of intimate partner violence on the SF-12 scores

Type of abuse	Physical component summary (*n*= 2177)			Mental component summary (*n*= 2177)		
		
	Effect*	SE	*P*	Effect*	SE	*P*
Psychological only	−1.38	0.79	0.083	−3.97	0.79	<0.001
Physical and/or sexual	−0.48	1.26	0.705	−4.97	1.25	<0.001
No abuse	0	—	—			

* Adjusted by demographics, socio-economic status, chronic illness in family, and in-law conflict.

There were no significant differences between the abused and nonabused women in terms of weeks of gestation at delivery, mode of delivery, birthweight, Apgar scores and admission to neonatal ICU (Table 6).

**Table 6:** Obstetric outcomes

	Nonabused (*n*= 2949)	Abused (*n*= 296)	*P* value
Gestation at delivery (weeks)	39.2 (1.3)	39.1 (1.3)	0.68
Birthweight (g)	3232 (414.9)	3242 (407.2)	0.71
Apgar score at 5 minutes	9.3 (0.7)	9.3 (0.7)	0.84
Normal delivery, *n* (%)	1816 (65.6)	187 (65.6)	0.62
Admission to neonatal ICU, *n* (%)	197 (7.1)	28 (9.9)	0.09

Values are presented as mean (SD) unless otherwise indicated.

## Discussion

This is the first population-based study conducted in Hong Kong to examine the impact of psychological abuse by an intimate partner on the mental health of pregnant women. The results support our hypothesis that psychological abuse against pregnant women has a negative impact on their mental health postdelivery. Our findings are consistent with those of a previous study in a similar local clinical population.^6^ An association between intimate partner violence and adverse mental health outcomes among pregnant women has been described in prior research studies involving non-Chinese participants.^17^–^21^ However, in our study, psychological abuse alone (rather than physical and/or sexual abuse as in earlier studies) was associated with higher depression scores. Given that preservation of face and maintenance of harmonious relationships are highly valued in Chinese culture,^22^ it is possible that Chinese women are more vulnerable to the effect of psychological abuse since the latter causes a loss of face and disharmony between the couple. Further investigation into the vulnerability of ethnic groups to different types of intimate partner violence is warranted.

In the present study, around three-fourths of the abuse reported was psychological only. This is comparable to the findings of previous local studies conducted in similar clinical populations in the past decade.^6^,^10^,^23^ Further studies are needed to ascertain whether intimate partner violence in Chinese couples is predominantly psychological in nature and to what extent culture influences the form of aggression in couple relationships. Psychological abuse almost always precedes physical abuse.^24^ It is possible that the psychological abuse experienced by those in the psychological abuse only group could be a precursor of physical abuse, a possibility given that more than half of the women in the physical and/or sexual abuse group also experienced psychological abuse. Pregnancy offers a window of opportunity for health professionals to help women who are psychologically abused to understand the devastating effects of ridiculing and shaming. Given the suggestion that progression from psychological to physical aggression can be as short as 12 months,^25^ the need for timely intervention is obvious.

The importance of using a culturally sensitive tool to assess intimate partner violence is clearly demonstrated in this study. Had we not clearly identified and explained psychological abuse using relevant examples as a separate item in the assessment, a large portion of the abuses would not have been detected. Even though it is said that abuse by an intimate partner is a family shame in the Chinese culture and would not be disclosed to outsiders,^26^ we have found that abused women were prepared to discuss their relationship problems with our research nurses. This underscores the importance of using well-trained and skilled practitioners when conducting research on a taboo subject.

We did not observe a higher risk of postnatal depression or thinking about harming oneself in the physical and/or sexual abuse group. While the relatively small number in this group may account for the observation, previous studies have found that for many women, frequent psychological abuse may be more damaging than an occasional physically abusive incident.^27^,^28^

Follingstad *et al.*^27^ offer an explanation for the detrimental effect of psychological abuse by identifying six types of emotional abuse patterns. Ridicule, as a form of emotional abuse, is the worst, as it attacks the woman's self-esteem and makes her feel worthless. By ridiculing the woman's traits (e.g. an attack on her character), her sense of hope and security in the relationship may be seriously damaged, leading to depression and low self-esteem. Such an assertion has been supported by other studies.^3^,^29^ In one study, abused women rated ridiculing of traits as the most severe form of psychological abuse and were more likely to take this personally as a direct attack on the self.^29^ In another study, shame, an affective reaction involving negative evaluations of the self with a corresponding sense of worthlessness, was found to play a vital role in the link between psychological abuse and post-traumatic stress disorder.^3^ Other forms of psychological abuse including threat of abuse, threat of divorce, jealousy, restriction and damage to property also contribute to the adverse effect of psychological abuse by evoking fear in and exerting coercive control over the victims.^27^ In this study, the most frequently cited examples of psychological abuse (‘shaming in front of friends/family’, ‘put-downs regarding appearance or behaviour’ and ‘ridiculing’) suggested that attacks on the women's self by their abusers were common. This may also explain the adverse impact of psychological abuse on the women's mental health independent of physical and/or sexual abuse.

It is possible that depression might, through cognitive bias, increase the likelihood of women perceiving psychological abuse in their relationship. Considering that the most frequently cited examples of psychological abuse (e.g. shaming in front of friends/families, ridiculing) are inherently subjective, it is plausible that depressed women may be more likely to perceive or interpret specific events as psychological abuse. As we did not ask the women about history of depression or treatment for depression, we were unable to decide if depression preceded the current pregnancy.

In addition to intimate partner violence, the participants in this study also reported other risk factors that are known to be associated with poor mental health, namely, low family income, debt and in-law conflict. More detailed qualitative and quantitative investigation is needed to explicate the interaction of the different risk factors on abused women's mental health.

Among the risk factors identified in this study, unplanned pregnancy has been reported in previous studies by the researchers.^6^,^10^,^23^ This is an interesting finding as Hong Kong has, over the years, developed successful reproductive health programmes that enable most women to control their fertility. It is apparent that, in at least some abusive households, the contraceptive needs of women are unmet. It has been suggested that the climate of fear and control surrounding abusive relationships could limit women's ability to control their fertility leading to unintended pregnancy.^30^ We support the recommendation^30^ that further investigation be conducted to explore the mechanisms through which intimate partner violence is associated with unintended pregnancy.

The findings, which are based on participants’ self-reports of socially stigmatised behaviours, are prone to recall bias and underestimation. The Chinese AAS, while more culturally sensitive for the study population than the original AAS, is limited to measuring certain types of abuse and does not investigate the context in which the abuse takes place. As such, examining abuse out of context may miss vital aspects of the entire situation.^31^ This study could have benefited from additional information concerning the intimate partner violence reported, such as, social worker, police or partner's reports. However, since most of the abuse was nonphysical, involvement of a social worker or the police would be unlikely. Also, involving the partner in the study may put the woman at greater risk for further abuse and would have been unethical given our promise to maintain confidentiality.

We interviewed the participants by telephone at 1 week postdelivery because they would only stay in hospital for 2–3 days and telephone was the only means by which to contact them. However, the women may not like responding to questions of abuse and mental health over the telephone, thus compromising the quality of the data. Future research might consider interviewing women face-to-face before discharge from hospital. Finally, the reported depression scores at 1 week following childbirth may be indicative of unresolved antenatal depression, ‘baby blues’ or postnatal depression. Future research should include a structured diagnostic interview for an assessment of the underlying psychopathology.

The lack of adverse effect of intimate partner violence on pregnancy outcomes in terms of miscarriage, preterm labour, fetal distress and low birthweight might have been expected but psychological aggression between parents can have extremely negative effects on children. Correlational research indicates that interparent verbal conflict and aggression predict a range of behavioural problems in children including aggression, delinquent behaviour, depression, anxiety, and poor social and academic competence.^28^ Thus, intervention is needed not only to reduce the harm on the mothers but also as a preventative measure for the children of abusive partner relationships.

Although difficult to organise, group work where experiences of psychological abuse can be shared, emotions validated and mutual support promoted can be considered. There is some evidence that group work can help to reduce the adverse effects of psychological abuse by increasing abused women's self-esteem and a sense of inner control.^32^

The association of intimate partner violence and adverse mental health outcomes has implications for clinical practice. Much has been written on good practice for working with women survivors of intimate partner violence with mental distress,^33^,^34^ including the need to offer safe choices to women experiencing intimate partner violence where mental health issues are also present, adopt a descriptive rather than interpretive approach during assessment, and promote collaborative partnerships among maternity, domestic violence and mental health professionals.

## Conclusion

This study found that psychological abuse by an intimate partner against pregnant women had a negative impact on their mental health postdelivery. This underscores the importance of screening pregnant women for intimate partner violence and the need for developing, implementing and evaluating interventions to address psychological abuse.
